# 84617 Prevalence and Co-prevalence of Comorbidities among Patients with Type 2 Diabetes Mellitus living in Puerto Rico, USA

**DOI:** 10.1017/cts.2021.488

**Published:** 2021-03-30

**Authors:** Enid Garcia-Rivera, Krystel Ruiz, Edgar Miranda, Luis Mejã, Cecile Marques, José Quijada, Homero Monsanto, Juan Orengo, Adolfo Pinzón

**Affiliations:** 1 University of Puerto Rico School of Medicine; 2 Merck & Co.; 3 MSD Chile

## Abstract

ABSTRACT IMPACT: Summarize the burden of diabetes comorbidities and its impact in healthcare utilization in Puerto Rico OBJECTIVES/GOALS: To estimate the prevalence of common comorbidities and describe the healthcare utilization patterns in patients with type 2 diabetes mellitus (T2DM) in Puerto Rico. METHODS/STUDY POPULATION: This is a descriptive study using healthcare claims data from patients with T2DM (based on ICD-9 diagnosis code) from most public and private healthcare insurance companies providing services in Puerto Rico in 2013 (representing more than 90% of insured population). Descriptive analyses by age, sex, type of insurance, health region, and type of medical encounter were done using frequency and percent for categorical data or means or median (with corresponding standard deviation or interquartile range) for continuous variables RESULTS/ANTICIPATED RESULTS: A total of 3,100,636 claims were identified from 485,866 adult patients with T2DM. Most patients were women (276,400; 57%), older than 65 years (235,390; 48%), from the Puerto Rico health regions of Caguas (79,604; 16%), Metro (66,280; 14%), or Bayamon (62,673; 13%) with private health insurance (371,806; 77%). The number of claims per patient ranged from 1 to 339. A mean of 6.3 claims (SD ±9.99) and a median of 3 claims (Q1 1- Q3 8) per subject were identified. Most (74%) were related to the diagnosis of diabetes (1,829,2015; 59%) or to cardiovascular diseases (458,219; 15%) and associated to outpatient services (2,722,727; 88%). The most prevalent comorbidities were hypertension (235,277; 48%), hyperlipidemia (197,449; 41%), neuropathy (100,471; 21%); renal disease (71,517; 15%), and retinopathy (61,837; 13%) DISCUSSION/SIGNIFICANCE OF FINDINGS: A high prevalence of comorbidities and use of healthcare services were identified in patients with T2DM, especially in older adults. Most comorbidities were due to diabetes-related conditions, highlighting the importance of early diagnosis and adequate management of T2DM patients to avoid preventable burden to the patient and the healthcare system

